# Risk Factors for Symptoms in Patients With Heterotopic Gastric Mucosa in the Upper Esophagus

**DOI:** 10.1155/grp/7658517

**Published:** 2025-01-09

**Authors:** Zhenxiang Wang, Ying Chen, Huihui Sun, Jie Xiong, Yu Zeng, Ye Chen, Yan Zhang, Zhiyu Dong, Junwen Wang, Guangbing Duan, Bo Li, Xue Qian, Kejing Sun, Tingting Zhan, Yuanxi Jiang, Shuchang Xu

**Affiliations:** ^1^Department of Gastroenterology, Tongji Institute of Digestive Diseases, Tongji Hospital, School of Medicine, Tongji University, Shanghai, China; ^2^Department of Pathology, Tongji Hospital, School of Medicine, Tongji University, Shanghai, China

**Keywords:** endoscopy examination, heterotopic gastric mucosa, inlet patch, mean nocturnal baseline impedance

## Abstract

**Goal:** This study is aimed at comparing the clinical characteristics and histological types of symptomatic and asymptomatic patients with heterotopic gastric mucosa in the upper esophagus (HGMUE) and exploring the factors influencing the occurrence and severity of laryngopharyngeal reflux (LPR) symptoms in these patients.

**Background:** HGMUE is a potential cause of LPR symptoms.

**Study:** This retrospective analysis evaluated 70 patients with HGMUE using a detailed questionnaire. Clinical, histological, high-resolution manometry, and 24-h pH impedance monitoring data were compared between symptomatic (*n* = 49) and asymptomatic (*n* = 21) patients.

**Results**: The diameter of HGMUE was significantly larger in the symptomatic group (*p* < 0.05), and the incidence of LPR symptoms increased with larger diameter grades; male patients were more likely to have LPR symptoms. The incidence of LPR symptoms varied significantly across histological classifications, being highest in patients with the fundic type (*χ*^2^ = 6.64, *p* < 0.05). Binary logistic regression analysis identified sex and histological type as risk factors for LPR symptoms, with odds ratios of 8.996 (95% confidence interval (CI): 1.350–59.962) and 8.493 (95% CI: 1.486–48.522), respectively. The mean nocturnal baseline impedance (MNBI) in the upper esophagus was significantly lower in the symptomatic group (1676.82 ± 739.09 *Ω* vs. 2441.01 ± 604.11 *Ω*; *p* < 0.05). Clinical and demographic characteristics did not significantly affect the severity of LPR symptoms.

**Conclusion:** The diameter, histological type, and sex of patients are risk factors for the occurrence of LPR symptoms in patients with HGMUE. More attention should be paid to patients with these factors. The MNBI is an effective indicator of the symptoms and treatment.

## 1. Introduction

Heterotopic gastric mucosa in the upper esophagus (HGMUE), also known as an “inlet patch,” refers to gastric mucosa located ectopically in the upper esophagus. Endoscopically, it appears as an orange–red mucosal island, typically single, though it can also be multiple [1]. The detection rate of HGMUE by endoscopy varies across different regions [2, 3]. Previous studies suggest that narrow-band imaging (NBI) mode significantly increases the detection rate of HGMUE [4]. The etiology of HGMUE remains unclear; some studies suggest it results from a congenital anomaly of the local esophageal mucosa, while others propose that its mechanism is similar to that of Barrett's esophagus [1, 5]. HGMUE presents several factors that warrant further investigation. However, the relationship between HGMUE and laryngopharyngeal reflux (LPR) symptoms remains controversial.

Previous clinical studies have explored whether HGMUE is associated with LPR symptoms, and most have concluded in the affirmative [6–11]. Some patients need to receive medication or endoscopic treatment due to significant LPR symptoms, which has a great impact on patients' lives [12–15]. However, not all patients with HGMUE have clinical symptoms, there is a variable prevalence of LPR symptoms reported for this condition that ranges from 20% to 73.1% [1, 7, 16]. And the severity of symptoms varies among symptomatic patients.

Most previous clinical studies have analyzed the differences in clinical characteristics and incidence of LPR between patients with HGMUE and those without. There has been no analysis of the presence or severity of symptoms in patients with HGMUE, and it is of great significance to guide the treatment and prognosis of patients in clinical practice. In recent years, studies have proposed a new impedance index of the 24-h pH impedance monitoring: the mean nocturnal baseline impedance (MNBI) [17]. MNBI can reflect the acid load of the local mucosa by indicating the mucosal integrity and can be effectively used to evaluate treatment and prognosis in patients with gastroesophageal reflux disease (GERD) [18].

Therefore, this study is aimed at combining demographic and other clinical characteristics to identify the factors influencing the occurrence and severity of LPR symptoms in patients with HGMUE and further reveal the relationship between LPR symptoms and acid secretion function of HGMUE through MNBI.

## 2. Materials and Methods

### 2.1. Patient Enrollment

Seventy patients diagnosed with HGMUE by endoscopy and confirmed by pathology between November 2019 and January 2021 were collected. LPR symptoms in these patients were investigated by questionnaire, and the patients were divided into symptomatic (*n* = 49) and asymptomatic (*n* = 21) groups according to the results of the questionnaire. Patients had to be able to answer the questionnaire independently. The exclusion criteria included patients under 18 years old; patients with serious respiratory, cardiovascular, or cerebrovascular diseases; emergency endoscopy patients; patients with advanced esophageal tumor; patients after surgery; those who refused to respond to the questionnaire; and those with continuous use of proton pump inhibitors (PPIs) exceeding 2 weeks before the endoscopy. This study received approval from the Research Ethics Committee of the Shanghai Tongji Hospital.

### 2.2. Endoscopic Examination

All patients who underwent endoscopy at our hospital signed an informed consent form before the examination. Each patient was examined by experienced endoscopists, and careful observation was conducted in alternating white light mode and NBI mode when the endoscope was stepped back to the esophagus. The HGMUE was observed as circular or oval orange-red island mucosa with a distinct border in white light mode, whereas the lesions were brown in the NBI mode. After HGMUE was identified by endoscopy, its maximum diameter (centimeters), distance to incisors (centimeters), number, and presence of reflux esophagitis (RE) were recorded. The diameter of HGMUE was determined by the top span of the fully open biopsy forceps. We classified the size by using the maximum diameter and divided the HGMUE into three grades: small (0–0.5 cm), medium (0.6–1.0 cm), and large (> 1.0 cm). One or two biopsies were obtained from each patient with HGMUE for histological examination.

### 2.3. Questionnaire

All the patients with HGMUE were carefully assessed by trained endoscopists regarding any LPR symptoms experienced during the previous 3 months. The symptoms evaluated included hoarseness, clearing throat, excess throat mucus, difficulty swallowing, coughing after eating or lying down, difficulty breathing, annoying cough, global sensation, and heartburn. We used the reflux symptom index (RSI) to assess and assist patients in evaluating the severity of symptoms using scores of 0–5 (0: *no symptoms*; 1: *symptoms are mild and can be ignored*; 2: *symptoms are mild but cannot be ignored*; 3: *symptoms are obvious but do not affect lif*e; 4: *symptoms affect daily life*; 5: *symptoms are very severe*). The RSI was used to diagnose LPR with an overall score > 13 [19]. Patients were classified into symptomatic and asymptomatic groups according to the existence of LPR symptoms. In this study, 13 was used as the threshold, with scores > 13 defined as severe and scores < 13 as mild. The patients' history of smoking, alcohol consumption, diabetes, and hypertension were also recorded.

### 2.4. Histological Assessment

Biopsy tissues were sectioned and stained using hematoxylin and eosin, and all histological tissues were examined by professional pathologists to identify their histological types. The histology of HGMUE was classified based on the contents of parietal and chief cells as follows: fundic (mucosal gland contained chief cells and parietal cells), transitional (mucosal gland contained mucinous cells and a few parietal cells), and antral (mucosal gland only contained mucinous cells) types [20–22]. The fundic and transitional types were classified as oxyntic because of the existence of parietal cells, whereas the antral type was classified as nonoxyntic.

### 2.5. Esophageal High-Resolution Manometry and 24-h Multichannel Intraluminal Impedance pH-Metry

Some patients who were diagnosed with HGMUE and completed the questionnaire assessment were given an appointment for esophageal high-resolution manometry and multichannel impedance pH monitoring examination (Medical Measurement Systems (MMS), the Netherlands) based on their willingness to participate. In esophageal high-resolution manometry, a silicone pressure catheter was inserted via the nasal cavity to the stomach, its location was adjusted, and the upper esophageal sphincter (UES) and lower esophageal sphincter (LES) were defined. The mean pressures of the UES and LES and the distal contractile integral (DCI) were recorded to test the esophageal motility. In multichannel impedance pH-metry, localization of LES was defined using the manometry test. The impedance pH catheter was placed in the esophagus, and the pH sensor was positioned 5 cm above the LES, with the rest of the impedance sensors located at 1, 3, 5, 7, 9, 13, 15, and 17 cm above the pH sensor. Patients were asked to conduct their normal activities of daily life and record their symptoms. The MNBI of the most proximal impedance channel (approximately 18 cm to the incisor) was calculated during the nighttime recumbent period. Three 10-min periods (~1:00 AM, 2:00 AM, and 3.00 AM) were selected, excluding refluxes, swallows, and pH drops. The mean of the three measurements was calculated to obtain the MNBI. Acid exposure time (AET) was also collected, and AET ≥ 6.0% is recognized as histological acid reflux as described in the Lyon Consensus [17].

### 2.6. Statistical Analysis

All statistical analyses were performed using the Statistical Package for the Social Sciences (SPSS) 13.0 software (International Business Machines (IBM), Armonk, New York, United States). Categorical variables were compared using the *χ*^2^ test or Fisher's exact test, and continuous variables were compared using Student's *t*-test and univariate analysis. *p* < 0.05 was considered statistically significant. In a binary logistic regression, the presence of laryngeal reflux symptoms was chosen as the outcome variable. The odds ratios and their corresponding 95% confidence intervals (CIs) served to describe the strength of the influence exerted by the retained predictor variable in the multivariate model.

## 3. Results

### 3.1. Demographic Characteristics of Symptomatic and Asymptomatic Groups

From November 2019 to January 2021, 70 patients (men: 44 (62.9%); women: 26 (37.1%)) with HGMUE were included. Forty-nine patients were classified as symptomatic, whereas 21 comprised the asymptomatic group. The proportion of symptomatic males was significantly higher than that of females (81.8% vs. 50.0%; *p* < 0.01), but there were no between-group differences regarding age, and history of smoking, alcohol consumption, hypertension, and diabetes (Table 1).

### 3.2. Differences in Endoscopic Characteristics Between Symptomatic and Asymptomatic Groups

The mean maximum diameter of HGMUE in the symptomatic group (1.09 ± 0.80 cm) was significantly larger than that in the asymptomatic group (0.67 ± 0.49 cm); *p* < 0.05. According to the classification premise, the proportion of symptomatic patients with large-diameter HGMUE was 82.8%, and the proportions with medium- and small-diameter lesions were 71.4% and 38.5%, respectively; the differences were significant (*p* < 0.05). However, the distance to the incisor, the number of HGMUE, and the presence of accompanying RE exhibited no between-group differences (Table 1).

### 3.3. Association Between Histological Type and LPR Symptoms

There are three main histological types of HGMUE. In our study, the biopsies of 35 patients revealed fundic type (50.0%), 15 (21.4%) were transitional, and 20 (28.6%) were antral. A total of 50 patients were classified as oxyntic type, and the others were nonoxyntic. The proportion of symptomatic patients in the oxyntic type was significantly higher than in the nonoxyntic type (79.2% vs. 50.0%; *p* < 0.05), and the proportions also showed significant differences in the three histological types. The proportion tended to increase from the antral type to the fundic type, which represents the increasing numbers of parietal cells (Table 1).

### 3.4. Binary Logistic Regression Analysis of Factors Associated With LPR Symptoms

Patient's gender, history of smoking and alcohol consumption, size and number of HGMUE, histological types, and whether accompanied by RE were included for binary logistic regression analysis. The results revealed that sex and histological types are risk factors for the occurrence of LPR symptoms in patients with HGMUE. The odds ratio for developing symptoms was 8.996 (95% CI 1.350–59.962) in relation to male and 8.493 (95% CI 1.486–48.522) regarding the fundic type (Table 2).

### 3.5. Endoscopic and Pathological Characteristics of Different Genders

We noted gender differences in HGMUE symptoms. Consequently, further analyses of endoscopic and pathological features were conducted on the basis of gender. The results suggested that there was no significant difference in the type of pathology between genders. The maximum diameter of HGMUE was longer in male patients, although there was no statistical difference. However, the proportion of multiple HGMUE was higher in male patients compared to female patients (Table 3).

### 3.6. Analysis of Factors Affecting Symptom Severity and Number of Symptoms

The severity of LPR symptoms varies among patients. The scores of the nine kinds of symptoms assessed by patients themselves using the RSI questionnaire were summed to evaluate symptom severity. Of the 49 symptomatic patients, 41 (83.7%) scored RSI < 13 points, and 8 patients had RSI ≥ 13. There were no differences in RSI scores between the sexes, numbers of HGMUE, history of smoking, alcohol consumption, hypertension, and the existence of parietal structures (Table 4). Symptomatic patients were subsequently divided into two groups according to symptom severity: RSI < 13 as mild and RSI ≥ 13 as severe. No statistically significant between-group differences regarding the size of HGMUE (*p* = 0.96) and histological types (*p* = 0.82) were observed between these two groups (Table 5).

**Figure 1:**
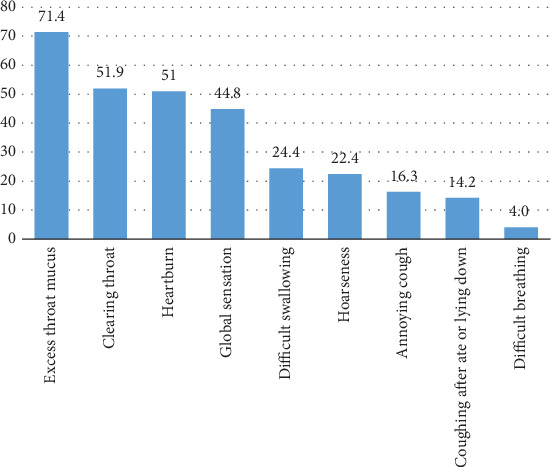
Proportions of each type of laryngeal reflux symptom in patients with HGMUE. HGMUE, heterotopic gastric mucosa in the upper esophagus.

According to the data of the RSI questionnaire, the proportion of excess throat mucus was highest in the 49 symptomatic patients, followed by clearing throat, heartburn, and global sensation (Figure 1). We defined cases with one kind of symptom as single symptom cases and those with more than one as multiple symptom cases. However, the data did not reveal differences between the two groups with respect to the number, size of HGMUE, and histological types (Table 6).

### 3.7. Comparison of High-Resolution Manometry and 24-h Impedance pH-Metry Characteristics Between Symptomatic and Asymptomatic Groups

Of the 70 patients, 23 underwent high-resolution manometry and 24-h impedance pH-metry. According to the Lyon Consensus, there were three cases with AET ≥ 6.0% which is defined as histological acid reflux. To exclude the influence of histological gastroesophageal reflux on manometry and esophageal impedance in patients with HGMUE, cases with AET ≥ 6.0% were excluded from this study. The manometry data indicated that the mean pressure of the UES (25.65 mmHg vs. 39.10mmHg, *p* = 0.59), LES (24.45 mmHg vs. 15.20 mmHg, *p* = 0.10), and mean DCI value (629 mmHg·s·cm vs. 775 mmHg·s·cm, *p* = 0.76) exhibited no significant differences in the symptomatic and asymptomatic groups (Table 7).

**Figure 2:**
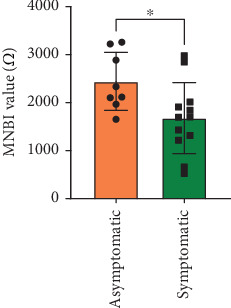
Differences in MNBI based on the presence or absence of symptoms. MNBI, mean nocturnal baseline impedance. *p* value is 0.03.

**Figure 3:**
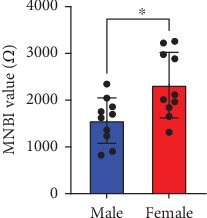
Differences in MNBI based on the gender. MNBI, mean nocturnal baseline impedance. *p* value is 0.01.

Because HGMUE is usually located in the esophagus about 18 cm away from the incisors, in this study, the MNBI of the uppermost channel was considered to reflect the acid load of the local mucosa. According to the study by Frazzoni et al. [23], the cut-off value of MNBI in healthy individuals is 2292 *Ω*, and we classified patients into lower and normal groups. In our study, the MNBI in symptomatic cases (1676.82 ± 739.09 *Ω*, *n* = 12) was significantly lower than the normal cut-off value, and the MNBI in asymptomatic cases (2441.01 ± 604.11 *Ω*, *n* = 8) was higher than the normal cut-off value. The MNBI of symptomatic cases was significantly lower than that of asymptomatic cases (*t* = 2.43, *p* < 0.05; Figure 2). Among patients with MNBI below the normal value (2292 *Ω*), 12 cases (85.7%) possessed parietal cells in the mucosal histological biopsy, and the proportion was higher than the 3 cases (50.0%) with normal MNBI values; however, the difference was not statistically significant (*χ*^2^ = 2.857, *p* < 0.13). Notably, male patients (1564.56 ± 484.78 *Ω*, *n* = 10) had significantly lower MNBI values than female patients (2323.43 ± 698.16 *Ω*, *n* = 10) (*t* = 2.82, *p* < 0.05; Figure 3).

## 4. Discussion

As a mucosal lesion, HGMUE has previously been reported to have a significant correlation with LPR symptoms. However, a proportion of patients have no obvious symptoms, and the severity of symptoms varies among the symptomatic patients. In this study, we found that gender and the diameter and histological type of HGMUE were significant risk factors for patients to have symptoms. The main type of LPR symptom in patients with HGMUE was excess throat mucus, and the RSI scale indicated that the severity of this symptom in the symptomatic patients was mild. MNBI further indicated that the symptoms of patients were related to the acid secretion function of HGMUE, which can be used as an indicator to guide treatment and prognosis.

Some previous studies included sectional analysis of symptoms when comparing the differences between HGMUE (−) and HGMUE (+) groups. Kazutoshi et al. found that the maximum length of the HGMUE was greater in the globus (+) group than in globus (−) group (1.23 ± 0.71 cm vs. 0.87 ± 0.58 cm) [3] and indicated that larger maximum diameters are more likely accompanied with globus but limited the symptoms to globus only. Most studies recorded and counted the number of HGMUE but did not further analyze its relationship with the symptoms. Previous studies revealed that HGMUE is often accompanied by RE and Barrett's esophagus. Yuksel et al. analyzed the endoscopic findings in HGMUE and found that the incidence of esophagitis was higher in such patients than in the general population (25.1% vs. 5.6%, *p* < 0.001) [24], but this difference was not analyzed with regard to the occurrence of symptoms. In this study, we use RSI to systematically evaluate patients' LPR symptoms and perform analysis by gathering the characteristics likely related to the occurrence of symptoms. The data indicated that the incidence of LPR symptoms was significantly higher in males than in females (81.8% vs. 50%). Initially, we thought that this difference was related to more frequent smoking and drinking in men, but there was no significant difference in the incidence of LPR symptoms in patients with HGMUE with a history of smoking and drinking. Further analysis of the data revealed that the proportion of multiple HGMUE was higher in male patients compared to female patients. The maximum diameter of HGMUE was longer in male patients, although there was no statistical difference and male patients have lower MNBI. The results indicate that, despite the absence of a statistically significant difference in histological type, male patients exhibited a greater tendency to present with larger and multiple HGMUE, which resulted in a heavier local mucosal acid load. The mean maximum diameter in the symptomatic group is larger than that in the asymptomatic group (1.09 ± 0.80 cm vs. 0.67 ± 0.49 cm, *p* < 0.05). It is suggested that the larger the maximum diameter, the more likely the patients are to exhibit symptoms. There were no differences regarding the age, history of smoking, alcohol consumption, hypertension and diabetes, number, distance to incisor, and accompanying RE. Interestingly, patients with multiple lesions appeared more likely to be symptomatic, but this was not confirmed in this study.

One of the most interesting features of HGMUE is the ability to secrete acid. In 1993, Nakajima et al. first discovered that the pH was lower at the HGMUE lesion [25]. Subsequently, some studies confirmed this phenomenon using 24-h double-channel pH-metry [8, 26]. The fundic and transitional histological types of HGMUE contain parietal cells and are generally located in the upper esophagus, closer to the throat which lacks the protective mechanism against gastric acid–pepsin, thereby providing a structural basis for the origin of symptoms. However, no studies have thus far analyzed the relationship between the ability to secrete acid and the occurrence of LPR symptoms. In our research, the proportion of symptomatic patients is higher in the parietal cell (+) group, and the proportion exhibits an increasing trend from antral to transitional to fundic types. The result indicated that the LPR symptoms are related to the existence of parietal cells and are also impacted by the quantity of parietal cells in HGMUE. Further binary logistic analysis indicated that the histological type is a risk factor for the occurrence of LPR symptoms (OR = 8.493, 95% CI 1.486–48.522).

Currently, 24-h pH impedance monitoring is considered effective for monitoring reflux events and acid load. In 2018, the “Lyon Consensus” introduced MNBI as a sensitive indicator for impedance monitoring [17]. Patel et al. found that the distal esophageal MNBI value was negatively correlated with AET and could independently predict the efficacy of antireflux therapy [18]. There are also related studies suggesting that MNBI can distinguish LPR and predict the treatment efficacy of LPR [27]. Therefore, we selected MNBI to determine the acid load of the mucosal site in HGMUE. In this study, the local mucosal MNBI value of the symptomatic group was significantly lower than that of the asymptomatic group. This result is in agreement with what was concluded in the previous section.

In clinical practice, the severity of LPR symptoms in patients with HGMUE varies among individuals. We used the RSI scale to analyze the number, type, and severity of LPR symptoms in patients. Among the symptomatic group in this study, there was no significant difference in the RSI score regarding demographic and endoscopic characteristics, as well as histological types. The results reveal that there were 41 (83.7%) patients with RSI < 13 points, and only 8 (16.3%) patients had RSI ≥ 13 points. We believe that because the symptoms in patients with HGMUE are relatively mild, the influence of factors such as the diameter, the number of HGMUE, and the type of pathology on the severity of symptoms is not effectively reflected. HGMUE can exhibit multiple symptoms, and there is no relevant clinical study exploring the factors affecting the type of symptoms. In this study, patients were subdivided into single- and multiple-symptom groups, but the number of symptoms showed no correlation with the number, histological type, or maximum diameter of HGMUE.

Other studies have shown that HGMUE is often accompanied by gastroesophageal reflux and abnormal esophageal motility. When the esophageal motility is abnormal, the esophageal propulsive clearance function is weakened, which could easily lead to excessive exposure to stimulation. Some esophageal manometry indexes were analyzed in this study but showed no difference between symptomatic and asymptomatic groups regarding the UES mean resting pressure, LES mean resting pressure, and the DCI. Based on the current data, no esophageal motility factors have been shown to significantly affect the occurrence of symptoms in patients with HGMUE.

In summary, LPR symptoms are quite common among patients with HGMUE. Most symptoms are mild but cannot be ignored. It should be noted that the study is limited by the inclusion of a relatively small number of patients and the inability to perform pH impedance monitoring on all patients, which may introduce patient and selection bias. However, the conclusion drawn from the analysis that the occurrence of LPR symptoms in HGMUE is closely related to gender, lesion diameter, and histological type, particularly the fundic type, may be still useful for further assessment of patients and aid in guiding treatment. Although most patients with HGMUE experience mild symptoms, patients with severe LPR symptoms should be actively treated for HGMUE, and MNBI can be used as an indicator to guide treatment and prognosis. Treatment of HGMUE by radio frequency/argon plasma coagulation (RF/APC) is recommended for symptomatic patients, and previous studies have suggested that both approaches are safe and effective [14, 15].

## Figures and Tables

**Table 1:** Demographic, endoscopic, and histological data and symptoms.

**Variables**	**Symptomatic (** **n** = 49**)**	**Asymptomatic (** **n** = 21**)**	**p** ** value**
Age (years) ($\bar{\chi }\pm s$)	50.80 ± 13.5	48.62 ± 12.3	0.53
Sex, *n* (%)			
Male	36 (81.8)	8 (18.2)	< 0.01⁣^∗^
Female	13 (50.0)	13 (50.0)	
History of smoking, *n* (%)			
Yes	19 (38.8)	4 (19.0)	0.10
No	30 (61.2)	17 (81.0)	
History of alcohol consumption, *n* (%)			
Yes	24 (49.0)	7 (33.3)	0.23
No	25 (51.0)	14 (66.7)	
History of diabetes, *n* (%)			
Yes	2 (4.1)	0 (0.0)	1.00
No	47 (95.9)	21 (100.0)	
History of hypertension, *n* (%)			
Yes	9 (18.4)	1 (4.8)	0.26
No	40 (81.6)	20 (95.2)	
HGMUE diameter (cm) ($\bar{\chi }\pm s$)	1.09 ± 0.8	0.67 ± 0.49	< 0.01⁣^∗^
Diameter grade, *n* (%)			
Small (*d* ≤ 0.5 cm)	5 (38.5)	8 (61.5)	0.01⁣^∗^
Medium (0.5 cm < *d* < 1.0 cm)	20 (71.4)	8 (28.6)	
Large (*d* ≥ 1.0 cm)	24 (82.8)	5 (17.2)	
Distance to incisor (cm) (*M*)	18.0 (17.5, 18.0)	18.0 (16.5, 18.0)	0.85
Number of HGMUE, *n* (%)			
Single	26 (72.2)	10 (27.8)	0.68
Multiple	23 (67.6)	11 (32.4)	
Reflux esophagitis, *n* (%)			
Positive	9 (81.8)	2 (18.2)	0.49
Negative	40 (67.8)	19 (32.2)	
Parietal cells, *n* (%)			
Negative	11 (50.0)	11 (50.0)	0.01⁣^∗^
Positive	38 (79.2)	10 (20.8)	
Histological type, *n* (%)			
Fundic	29 (82.9)	6 (17.1)	0.04⁣^∗^
Transitional	10 (66.7)	5 (33.3)	
Antral	10 (50.0)	10 (50.0)	

Abbreviation: HGMUE, heterotopic gastric mucosa in the upper esophagus.

⁣^∗^*p* < 0.05 was considered statistically significant.

**Table 2:** Factors associated with the occurrence of LPR symptoms by binary logistic regression analysis.

**Category**	**p** ** value**	**OR**	**95% CI**
Sex (male)	0.023⁣^∗^	8.996	(1.350, 59.962)
History of smoking (positive)	0.383	2.409	(0.334, 17.385)
History of alcohol consumption (positive)	0.268	0.317	(0.041, 2.419)
Diameter of HGMUE			
Diameter ≤ 0.5 cm	0.148		
0.5 cm < diameter < 1.0 cm	0.425	2.265	(0.304, 16.864)
Diameter ≥ 1.0 cm	0.070	6.689	(0.854, 52.403)
Number (multiple)	0.095	0.277	(0.061, 1.252)
Histological type			
Antral	0.055		
Fundic	0.016⁣^∗^	8.493	(1.486, 48.522)
Transitional	0.204	3.388	(0.516, 22.260)
Reflux esophagitis (positive)	0.352	2.885	(0.309, 26.907)

Abbreviations: HGMUE, heterotopic gastric mucosa in the upper esophagus; LPR, laryngopharyngeal reflux.

⁣^∗^*p* < 0.05 was considered statistically significant.

**Table 3:** Endoscopic and pathological characteristics of different genders.

**Variables**	**Male (** **n** = 44**)**	**Female (** **n** = 26**)**	**p** ** value**
Histological type, *n* (%)			
Fundic	23 (52.3)	12 (34.3)	0.69
Transitional	8 (18.1)	7 (46.7)	
Antral	13 (29.5)	7 (35.0)	
Parietal cells, *n* (%)			
Negative	13 (29.5)	7 (26.9)	0.81
Positive	31 (70.5)	19 (73.1)	
HGMUE diameter (cm) ($\bar{\chi }\pm s$)	1.05 ± 0.55	0.81 ± 0.45	0.06
Number of HGMUE, *n* (%)			
Single	18 (40.9)	18 (69.2)	0.03⁣^∗^
Multiple	26 (59.1)	8 (30.8)	

Abbreviation: HGMUE, heterotopic gastric mucosa in the upper esophagus.

⁣^∗^*p* < 0.05 was considered statistically significant.

**Table 4:** Clinical characteristics and severity of symptoms.

**Variables**	**RSI (M)**	**Z**	**p** ** value**
History of alcohol consumption			
Positive	10 (5, 12.8)	−1.26	0.21
Negative	7 (3, 9.5)		
History of smoking			
Positive	8 (5, 12)	−1.16	0.25
Negative	7 (3, 10)		
History of hypertension			
Positive	5 (2, 7.5)	−1.76	0.08
Negative	8.5 (5, 11)		
Sex			
Male	7.5 (5, 11)	−0.41	0.68
Female	6 (3, 10.5)		
Number of HGMUE			
Single	8 (4.5, 11)	−0.15	0.88
Multiple	7 (3, 11)		
Parietal cells			
Positive	7 (3, 10)	−0.71	0.48
Negative	8 (5, 12)		

*Note:p* < 0.05 was considered statistically significant.

Abbreviations: HGMUE, heterotopic gastric mucosa in the upper esophagus; RSI, reflux symptom index.

**Table 5:** Histological type, maximum diameter, and severity of symptoms.

**Variables**	**RSI grade**			
	**< 13 (** **n** = 41**)**	**≥ 13 (** **n** = 8**)**	**Total**	**p** ** value**
Histological type, *n*				
Fundic	24	5	29	0.82
Transitional	9	1	10	
Antral	8	2	10	
Diameter grade (*d*), *n*				
*d* ≤ 0.5 cm	4	1	5	0.96
0.5 cm < *d* < 1.0 cm	17	3	20	
*d* > 1.0 cm	20	4	24	

*Note:p* < 0.05 was considered statistically significant.

Abbreviation: RSI, reflux symptom index.

**Table 6:** Clinical factors and the number of symptoms.

	**Solitary symptoms (** **n** = 9**)**	**Multiple symptoms (** **n** = 40**)**	**χ** ^2^	**p** ** value**
Number of HGMUE, *n* (%)				
Single	3 (11.5)	23 (88.5)	1.72	0.27
Multiple	6 (26.1)	17 (73.9)		
Histological type, *n* (%)				
Fundic	5 (17.2)	24 (82.8)	0.33	1.00
Transitional	2 (20.0)	8 (80.0)		
Antral	2 (20.0)	8 (80.0)		
Diameter, *n* (%)				
*d* ≤ 0.5 cm	0 (0.0)	5 (100.0)	2.76	0.22
0.5 cm < *d* < 1.0 cm	6 (30.0)	14 (70.0)		
*d* ≥ 1.0 cm	3 (12.5)	21 (87.5)		

*Note:p* < 0.05 was considered statistically significant.

Abbreviation: HGMUE, heterotopic gastric mucosa in the upper esophagus.

**Table 7:** Relationship between high-resolution manometry data and symptoms.

**Variables**	**Symptomatic (** **n** = 12**)**	**Asymptomatic (** **n** = 8**)**	**Z**	**p** ** value**
Mean pressure of UES (mmHg)	25.65 (21.28, 47.70)	39.10 (19.5, 48.1)	−0.54	0.59
Mean pressure of LES (mmHg)	24.45 (17.10, 29.85)	15.20 (14.10, 19.18)	−1.66	0.10
DCI (mmHg·s·cm)	629 (197.50, 973.25)	775 (274, 1350.75)	−0.31	0.76

*Note:p* < 0.05 was considered statistically significant.

Abbreviations: DCI, distal contractile integral; LES, lower esophageal sphincter; UES, upper esophageal sphincter.

## Data Availability

The clinical data used to support the findings of this study are available from the corresponding author upon request.
